# Case report: Pediatric anti-gamma aminobutyric acid-B receptor encephalitis with benign prognosis

**DOI:** 10.3389/fped.2023.1104001

**Published:** 2023-03-03

**Authors:** Yeping Wang, Xiaoyan Ren, Yu Shen, Yi Hua, Lu Xu, Weiran Zhang, Guoxia Sheng, Peifang Jiang, Zhefeng Yuan, Liu Liu, Feng Gao

**Affiliations:** ^1^ Department of Neurology, Children's Hospital, Zhejiang University School of Medicine, National Clinical Research Center for Child Health, Hangzhou, China; ^2^ Department of Pediatric, Jinhua Maternity and Child Health Care Hospital, Jinhua, China; ^3^ Department of Neurology, Ningbo Women and Children’s Hospital, Ningbo, China

**Keywords:** anti-GABA_B_R encephalitis, autoimmune encephalitis, pediatric, benign prognosis, immunotherapy

## Abstract

**Objective:**

To explore the clinical characteristics of pediatric anti-gamma-aminobutyric acid-B receptor (GABA_B_R) encephalitis to enhance the understanding and improve the diagnostic and therapeutic strategies for this disease.

**Methods:**

We report a rare case of a female pediatric patient with anti-GABA_B_R encephalitis who was treated at the Children's Hospital of Zhejiang University School of Medicine. Literature search was performed to explore the clinical characteristics of pediatric anti-GABA_B_R encephalitis.

**Results:**

The patient exhibited recurrent epileptic seizure, status epilepticus, and psychiatric symptoms at the age of 11 years and 10 months. Anti-GABA_B_R antibodies were positive in cerebrospinal fluid and serum. Brain magnetic resonance imaging (MRI) exhibited abnormal signals in the left hippocampus. Symptoms and abnormality of brain MRI were improved after administration of immunosuppressants, anti-seizure and antipsychotic drugs. Two of pediatric anti-GABA_B_R encephalitis with clinical data were identified through literature search. Analysis of these three cases suggested that the pediatric patients primarily experienced limbic encephalitis, with no tumor incidence. A favorable immunotherapy response was demonstrated with a superior prognosis in all the cases.

**Conclusions:**

We reported a pediatric anti-GABA_B_R encephalitis case with early age of onset. Promt autoimmune antibody testing and tumor screening, as well as immunomodulatory treatment immediately after a definitive diagnosis are warranted to improve prognosis.

## Introduction

Autoimmune encephalitis (AE) broadly refers to a type of encephalitis that is mediated by autoimmune mechanisms, particularly by antineuronal antibodies such as those against neuronal cell surface receptors or synaptic proteins, and intracellular targets (1, 2). With the advancement of experimental antibody detection techniques and expansion of the neuronal antibody spectrum, an increasing number of antibody-related AE cases have been diagnosed in recent years.

GABA is a vital inhibitory neurotransmitter in the central nervous system that plays a critical role in controlling neuronal excitability by binding to specific receptors. GABA_A_, GABA_B_, and GABA_C_ receptors are the three classes of GABA receptors. Among these receptors, GABA_A_R and GABA_c_R are ligand-gated chloride channels, whereas GABA_B_R is a G-protein coupled receptor. However, GABA_B_R plays an essential role in neurotransmission and maintenance of synaptic stability (3). It is widely distributed in the brain and spinal cord, with the highest concentrations in the hippocampus, thalamus, and cerebellum. Therefore, it is closely associated with learning, memory, and cognitive functions (4).

Anti-gamma-aminobutyric acid-B receptor (GABA_B_R) encephalitis is a relatively rare type of AE. Additionally, most anti-GABA_B_R encephalitis cases have been reported in adults, and the disease has been rarely observed in children. Here we elaborate on the clinical manifestations of a case of pediatric anti-GABA_B_R encephalitis and analyze the clinical characteristics of the disease through literature review.

## Case presentation

**Figure 1:**
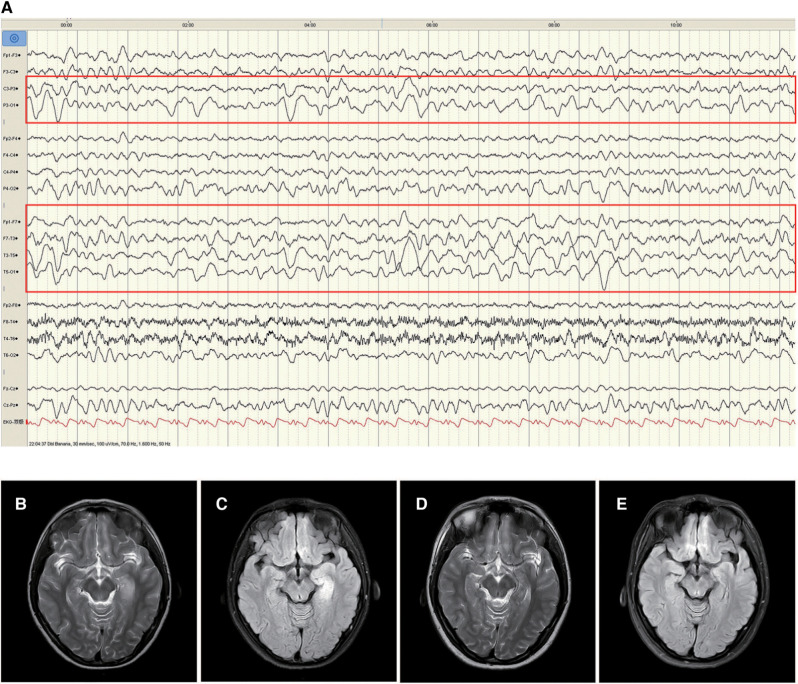
Electroencephalogram (EEG) and changes in brain magnetic resonance imaging (MRI) of patient 1. The left temporo-occipital polymorphic *δ* and *θ* activities were observed in the EEG of the patient in the waking phase at disease onset (**A**, red rectangle). MRI T2/fluid-attenuated inversion recovery (FLAIR) hyperintensities in the hippocampal gyrus in the left medial temporal lobe (**B** and **C**). Improvement of abnormal signal in MRI T2/FLAIR (**D** and **E**) three years after treatment.

Patient 1 was a 16-year-old girl, who experienced 2–3 episodes of seizure per day in sleep, with the onset age of 11 years and 10 months. The seizure was manifested as secondarily generalised tonic–clonic seizures that lasted 2–3 min before self-resolution. The patient experienced one episode of status epilepticus (SE) lasting 2 h. No abnormalities were observed in the medical, personal, or family history and upon physical examination. CSF test exhibited a white blood cell count of 30 × 106/L, comprising mainly monocytes (90%). No abnormalities were observed in CSF biochemistry, smear, and culture. Enteroviruses (hepatitis E virus, enterovirus type 71, coxsackievirus A16), Epstein–Barr virus, herpes simplex virus, and oligoclonal bands were negative in blood and CSF. Blood biochemistry, genetic metabolic profile, tumor markers, antinuclear antibodies, and erythrocyte sedimentation rate were normal. Video-electroencephalography (VEEG) indicated slowing of the basic rhythm in background activity during waking periods. Persistent polymorphic δ and θ rhythms were observed in the left temporo-occipital region during wakefulness and sleep (Figure 1A). The B-scan ultrasonography of the thyroid, liver, gallbladder, kidneys, retroperitoneum, and uterus; chest computed tomography (CT); electrocardiogram; and head magnetic resonance imaging (MRI) were normal. The patient experienced recurrent seizure incidence despite being symptomatically treated with acyclovir, mannitol, and levetiracetam (12.5 mg/kg·d). On day 23 after disease onset, brain MRI exhibited hyperintensities on T2 weighted image (T2WI) and fluid-attenuated inversion recovery (FLAIR) in the left medial temporal hippocampus (Figures 1B,C). Anti-GABA_B_R antibodies were positive in both CSF (1:3.2) and serum (1:32). However, antibodies against NMDAR, AMPAR, GABA_A_R, LGI1, and CASPR2 and paraneoplastic antibodies were not observed in the CSF or serum. The patient was then administered levetiracetam (25 mg/kg·d) combined with carbamazepine (10 mg/kg·d) to control epileptic seizures, followed by administration of methylprednisolone (10 mg/kg × 3d). Although the epileptic events reduced, symptoms such as disorganised speech, easily frightening, agitation, and auditory hallucinations appeared. Further treatment with methylprednisolone (18.75 mg/kg × 3d) combined with intravenous immunoglobulin (IVIG; 20 g × 3d) and sertraline (50 mg qn) was administered. The patient scored 3 on the mRS when the symptoms were most severe. Following treatment, the epileptic seizures ceased one month after onset, psychiatric symptoms disappeared, and the mRS score was 0. Re-examination of brain MRI indicated a slight improvement in abnormal hippocampal signals, which were significantly improved three years after disease onset (Figures 1D,E).

## Literature review

A literature search was conducted with the keywords ‘GABA_B_R' and ‘encephalitis’ in databases such as PubMed and CNKI until December 2022. Only the articles written in English or Chinese were included. Anti-GABA_B_R encephalitis cases of pediatric patients (aged less than 18 years) with complete or partial clinical data were identified, and the clinical characteristics of all these pediatric patients were summarized. And the patients with only anti-GABA_B_R-positive and not secondary to any infections were included in analysis.

The database search yielded seven pediatric anti-GABA_B_R encephalitis cases (5–10). In 2014, Kruer et al. (8) reported a case of anti-GABA_B_R encephalitis in a 3-year-old male patient. The patient exhibited severe clinical phenotypes and rapid progression manifesting as confusion, lethargy, opsoclonus, dystonic tongue movements, ataxia, and chorea at onset, followed by complex partial seizures and SE during later phases. Laboratory tests indicated positive anti-GABA_B_R antibodies in serum and CSF (1:32 and 1:3.2, respectively). A white blood cell count of 154/µl and a protein level of 59 mg/dl were observed in the initial CSF analysis. EEG exhibited generalised slow waves and epileptiform discharge. Brain MRI exhibited multifocal T2/FLAIR hyperintensities in the brainstem and cerebellum with involvement of the basal ganglia and hippocampus. Seizures were difficult to control even after treatment with various antiseizure medications (ASMs), intravenous immunogloblin (IVIG), and methylprednisolone. The patient died of sepsis four weeks later. The case of the same patient was reported by Petit-Pedrol et al. (11) in 2014; the patient tested positive for anti-GABA_A_R antibodies in CSF (1:320) and serum (+, titer was not reported) in addition to anti-GABA_B_R antibodies. However, the clinical features of this patient were highly consistent with anti-GABA_A_R encephalitis. Additionally, Liu et al. (9) reported a case of an 11-year-old male patient who experienced AE 24 days after having Japanese encephalitis, and the main clinical manifestations were fever, headache, irritability, and aggressive behaviour. Anti-GABA_B_R antibodies were positive in CSF and negative in serum. EEG and brain MRI findings were unknown. The patient displayed some mood problems even after immunotherapy. Neither of these two patients were recruited for analysis and discussion in consideration since they did not met the inclusion criteria. Another cohort study described a case of pediatric anti-GABA_B_R encephalitis in a 16-year-old patient, although no clinical data of the patient were reported (7). Kang et al. reported 103 antibody-positive AE patients, which including two patients with anti-GABA_B_R antibodies (10). These two patients were presented with epilepsy and behavioral symptoms. Abnormalities in EEG and brain MRI were also found. Regrettably, one of these two patients was positive for both anti-CASPR2 and anti-GABA_B_R antibodies, while the detailed clinical information of these two cases was difficult to distinguish from the paper. Therefore, these cases were also excluded from the analysis.

Jeffery et al. (6) and Höftberger et al. (5) each reported a case of a patient aged ≤18 years having anti-GABA_B_R encephalitis in their cohort studies, with partial descriptions of clinical data. These two patients were labelled Patient 2 and Patient 3, respectively. Both patients were aged 16 years at disease onset. Patient 2 presented with seizures, delirium, and psychosis, with a serum anti-GABA_B_R antibody titer of 1:1920; the symptoms were completely resolved after treatment with steroids and plasma exchange (PLEX). Patient 3 presented with typical limbic encephalitis (LE) (details unknown), with a positive anti-GABA_B_R antibody in serum; the symptoms were completely resolved after treatment with steroids, IVIG, and PLEX. Tumor was not observed in these two patients. The clinical characteristics of the three analyzed pediatric cases with anti-GABA_B_R encephalitis are summarized in Table 1. The mean age of disease onset was 14.6 years (11 years 10 months–16 years). All the three patients exhibited typical clinical symptoms of LE, tested negative in tumor screening, and displayed a good prognosis after immunotherapy.

**Table 1:** Clinical characteristics of three idiopathic pediatric patients with anti-GABA_B_R encephalitis.

	Sex	Onset Age	Clinical manifestation	CSF examination	Brain MRI	EEG	Anti-GABA_B_R	Treatment	Tumor	Outcome	Follow-up	Reference
1	F	11y10m	Seizure, SE, psychiatric symptoms	WBC 30 × 10^6^/L	T2/FLAIR: abnormal signals in the left hippocampus	Slowing of the basic rhythm in background activity during waking periods; persistent polymorphic *δ* and *θ* rhythms were observed in the left temporo-occipital region during wakefulness and sleep	Serum (1:32) CSF (1:3.2)	Steroids + IVIG + LEV, CBZ + Sertraline	N	Complete resolution	51 m	Present report
2	F	16y	Seizure, delirium, psychiatric symptoms	NA	NA	NA	Serum (1:1920) CSF (NA)	Steroids + PLEX	N	Complete resolution	14 m	Jeffery et al, 2013
3	F	16y	LE (no detail)	NA	NA	NA	Serum (+, NA) CSF (NA)	Steroids + IVIG + PLEX	N	Complete resolution	18 m	Höftberger et al, 2013

CBZ, carbamazepine; CSF, cerebrospinal fluid; EEG, electroencephalogram; F, female; FLAIR, fluid-attenuated inversion recovery; IVIG, intravenous immunoglobulin; LE, limbic encephalitis; LEV, levetiracetam; MRI, magnetic resonance imaging; N, No; NA, not available; PLEX, plasma exchange; SE, status epilepticus.

## Discussion

This case study reports a pediatric anti-GABA_B_R encephalitis case with the earliest onset age to date. The patient exhibited severe epileptic seizure, SE, psychiatric problems, behavioral abnormalities, elevated CSF cell counts, left hippocampal hyperintensities (as indicated on the T2WI and FLAIR MRI), and positive anti-GABA_B_R in serum and CSF. Immunotherapy was found to be an effective strategy. In addition, only seven pediatric anti-GABA_B_R encephalitis cases were identified through literature search, of which two cases with only anti-GABA_B_R-positive and not secondary to any infections, and their clinical characteristics were analyzed.

Fifteen cases of adult anti-GABA_B_R encephalitis were first reported by Lancaster et al. in 2010 (12). This disease is a GABA_B_R antibody-mediated AE that often involves the limbic system and exhibits the median onset age of 50–65 years on adults (Table 2, Supplementary data S1). The disease exhibits a male predisposition (60%–82%) in adults (13), with seizures as the first symptom followed by incidences of confusion and memory deficits, abnormal behavior, hallucinations, language disorder, sleep disorder, and cerebellar ataxia in a few cases (14–16). Epileptic seizures in patients with anti-GABA_B_R encephalitis are predominantly present as generalized tonic-clonie seizure (17–19). SE is common and displays a high propensity of progressing into refractory epilepsy when multiple ASMs are ineffective. The three pediatric cases summarized in this study presented with LE with epileptic seizures. SE occurred only in Patient 1, although its incidence in other two patients reported in literature is unknown due to lack of information. These clinical characteristics are similar to those reported in studies on adult anti-GABA_B_R encephalitis (Table 2).

**Table 2:** Differences between adults and pediatric patients with anti-GABA_B_R encephalitis.

	Sex	Median onset Age (y)	No. of cases	Manifestation	Cognitive /psychiatric symptoms	Refractory epilepsy	SE	EEG abnormalities	Brain MRI abnormalities	Tumor	Prognosis
Adults	M	50–65	501	LE	Y	Y	Y	Y	Y	50%–60%	Mostly good
Children	F	Juvenile	3	LE	Y	N	NA	NA	Y	Low	Good

EEG, electroencephalogram; F, female; LE, limbic encephalitis; M, male; MRI, magnetic resonance imaging; N, No; NA, not available; SE, status epilepticus.

Slow-wave activity was observed in the EEG of Patient 1 in this study with no information on the other two included cases. EEG abnormalities comprising predominantly slow waves, followed by epileptiform discharge (18–21) were detected in more than 80% of adult anti-GABA_B_R encephalitis cases (22, 23). Si et al. indicated that the distribution range of the slow-wave activity reflects the severity of anti-GABA_B_R encephalitis (24). In addition, changes in brain imaging can also facilitate the diagnosis and treatment of anti-GABA_B_R encephalitis. Signal anomalies in the unilateral or bilateral medial temporal lobe were observed in T2/FLAIR-weighted images of approximately 45% of patients with anti-GABA_B_R encephalitis (2), and some patients developed hippocampal sclerosis with disease progression (25). Furthermore, limbic system involvement was associated with a poor prognosis (26). In Patient 1 in this study, abnormalities were seen in T2/FLAIR-weighted images in the left hippocampus in early phase of the disease and were significantly reduced after treatment. Although the specificity of imaging manifestations is limited in the acute phase of anti-GABA_B_R encephalitis, long-term brain MRI follow-up can help clinicians in understanding the underlying pathological and physiological processes, thereby providing a theoretical basis for diagnosis and treatment.

Research suggests that other neuronal antibodies, including those against VGCC, AMPAR, and GABA_A_R and classic paraneoplastic antibodies (Hu, Ri, amphiphysin, SOX-1), could be detected in approximately 7%–40% of patients with GABA_B_R encephalitis (5, 19, 25), leading to variation and overlap of clinical syndromes. Although the Patient 1 was tested to rule out antibodies against NMDAR, AMPAR, GABA_A_R, LGI1, and CASPR2, a comprehensive screening against the entire set of known AE antigens was not performed, which is one of the limitations of this study. In the other aspect, tumor incidence was identified in the anti-GABA_B_R encephalitis population, as most of the patients were middle-aged or elderly people. Studies have revealed that 50%–60% of patients with anti-GABA_B_R encephalitis develop tumor. Small cell lung carcinoma has been the mostly commonly observed tumor, followed by thymic carcinoma, bladder cancer, and breast cancer (5, 27, 28). Furthermore, tumor progression is the most common cause of death in anti-GABA_B_R encephalitis (21). However, this type of AE is highly responsive to immunotherapy. No tumor was found in the three cases of pediatric anti-GABA_B_R encephalitis summarized in this study. Nevertheless, considering the correlation between the disease and tumor, susceptibility to tumor in pediatric patients with anti-GABA_B_R encephalitis cannot be ruled out. Tumor was identified after encephalitis in the majority of previous cohort studies. Therefore, long-term follow-up is needed for pediatric patients with anti-GABA_B_R encephalitis, despite negative results observed in the initial tumor screening. Graus et al. updated the diagnostic criteria for PNS, which recommend repeated tumor screening every 4–6 months for 2 years for patients with high-risk phenotypes (e.g., encephalomyelitis, LE, opsoclonus-myoclonus) and high-risk antibodies (e.g., Hu, CV2, SOX1, Yo), if no tumor is found in the initial examination (29).

Treatment for AEs mainly includes immunomodulation therapy, treatment of primary tumor, and symptomatic treatment. Encephalitis associated with antibodies against cell-surface proteins are mediated primarily by humoral immunity which is respond good to immunosuppression. Furthermore, if a tumor is identified, early tumor treatment is particularly crucial for a good outcome. Studies have suggested that most patients with anti-GABA_B_R encephalitis demonstrate complete or partial recovery of neurological functions after receiving immunotherapy (5, 15, 16, 30, 31). Moreover, immunotherapy delay is an independent predictor for epilepsy (32). Therefore, immunotherapy should be administered promptly after definitive diagnosis. Currently, the recommended first-line immunotherapies include treatment with steroid, IVIG, and PLEX, whereas second-line immunosuppressants such as rituximab and cyclophosphamide are generally used to treat refractory cases. All the three pediatric patients reported in this study received immunotherapy, did not develop tumor, and had a good outcome.

Given the low incidence of anti-GABA_B_R encephalitis and the rarer incidence of the disease in pediatric patients, there were three pediatric cases with only anti-GABA_B_R encephalitis could be analysed until now. Among of these cases, we reported a case with an early onset age of 11 years and 10 months. The phenotype of pediatric anti-GABA_B_R encephalitis is similar to that in adults, in whom the disease presents primarily as LE; however, tumors are rare in pediatric cases. With timely immunotherapy administration, the prognosis of pediatric patients may become superior to that of adult patients.

## Data Availability

The original contributions presented in the study are included in the article/**Supplementary Material**, further inquiries can be directed to the corresponding author/s.
